# Effects of Sacubitril/Valsartan on Blood Pressure and Proteinuria in Hypertensive Patients With Chronic Kidney Disease

**DOI:** 10.1111/jch.70089

**Published:** 2025-07-20

**Authors:** Maki Murakoshi, Takashi Kobayashi, Masao Kihara, Seiji Ueda, Yusuke Suzuki, Tomohito Gohda

**Affiliations:** ^1^ Department of Nephrology Juntendo University Faculty of Medicine Tokyo Japan; ^2^ Division of Kidney Health and Aging The Center for Integrated Kidney Research and Advance Shimane University Faculty of Medicine Shimane Japan

**Keywords:** chronic kidney disease, glomerular filtration rate, hypertension, proteinuria, sacubitril/valsartan

## Abstract

The effects of the angiotensin receptor–neprilysin inhibitor sacubitril/valsartan (Sac/Val) on blood pressure (BP) and proteinuria in patients with advanced chronic kidney disease and hypertension remain unclear. This retrospective study evaluated the effect of Sac/Val on BP, the urinary protein‐to‐creatinine ratio (UPCR), and the estimated glomerular filtration rate (eGFR) in 66 patients with hypertension and proteinuria (UPCR ≥ 0.15 g/g) who received renin–angiotensin system inhibitors at 1, 3, and 6 months. At baseline, the median eGFR and UPCR were 28.4 mL/min/1.73 m^2^ and 1.18 g/g, respectively. Significant reductions in systolic and diastolic BP, the eGFR, and the UPCR were observed over time (*p* values ranged from 0.03 to < 0.0001). At 1 month, 59% of patients showed a transient increase in the UPCR, and 21% had a ≥10% decline in the eGFR, with both metrics returning closer to baseline by 6 months. The percent change in the UPCR at 1 month was positively correlated with the percent change in the eGFR (*r* = 0.55, *p* < 0.0001). In conclusion, Sac/Val showed considerable BP‐lowering efficacy even in patients with impaired renal function and proteinuria. Early changes in the eGFR were positively correlated with changes in the UPCR, and patients with an early decline in the eGFR or an increase in proteinuria did not experience further worsening.

## 1 Introduction

Although initially developed for heart failure (HF), sacubitril/valsartan (Sac/Val) shows greater antihypertensive efficacy than valsartan alone [1]. In a phase III study involving Japanese patients with essential hypertension, Sac/Val showed superior efficacy over olmesartan and was approved for hypertension treatment in Japan in 2021 [2].

The PARADIGM‐HF trial showed that Sac/Val reduced cardiovascular mortality and HF hospitalizations by 20% compared with enalapril [3]. Furthermore, Sac/Val significantly slowed the decline in the estimated glomerular filtration rate (eGFR) compared with enalapril (−1.61 vs. −2.04 mL/min/1.73 m^2^/year, *p* < 0.001), although it was associated with a higher urinary albumin‐to‐creatinine ratio (UACR) at 1 and 8 months [4]. These findings suggest that Sac/Val confers renal benefits by improving cardiac function and modulating renal hemodynamics through the potentiation of natriuretic peptide (NP)–related hormones, which promote natriuresis and vasodilation [5, 6, 7]. This mechanism increases cyclic guanosine monophosphate levels, leading to afferent arteriole dilation, elevated renal blood flow, and potentially an improved eGFR [8]. However, this same mechanism may elevate intraglomerular pressure and proteinuria, potentially contributing to long‐term kidney damage. Therefore, while Sac/Val is expected to offer nephroprotective effects, its overall renal efficacy remains controversial. Additionally, most previous large‐scale studies on Sac/Val excluded patients with advanced chronic kidney disease (CKD), and studies that evaluated the effects of Sac/Val on proteinuria in patients with CKD have yielded inconsistent results [9, 10, 11]. Regarding the potential renoprotective effects of Sac/Val, examining early changes in glomerular hemodynamics following the initiation of treatment is important.

This study aimed to clarify the effects of switching from renin–angiotensin system (RAS) inhibitors to Sac/Val on blood pressure (BP), urinary protein excretion, and renal function including the eGFR in hypertensive patients with renal dysfunction and proteinuria. We particularly focused on early‐phase responses, especially those observed within 1 month after initiating Sac/Val treatment.

## 2 Methods

### 2.1 Patients

This retrospective analysis evaluated the effect of Sac/Val on BP and proteinuria in patients with hypertension and CKD. The study was conducted on patients taking RAS inhibitors (angiotensin‐converting enzyme inhibitors or angiotensin receptor blockers), which are standard treatments for hypertension in patients with proteinuria. This was an opt‐out study. This study was approved by the Ethics Committee of Juntendo University Hospital, with a waiver of the requirement to obtain informed consent (approval number: E24‐0175‐H01), and was conducted in accordance with the principles of the Declaration of Helsinki. Medical records were anonymized and de‐identified before the final analysis. Of the 129 patients with proteinuria (urinary protein‐to‐creatinine ratio [UPCR] ≥ 0.15 g/g) who were treated with Sac/Val for hypertension at Juntendo University Hospital between October 2021 and January 2024, 63 were excluded. The primary reasons for exclusion were the lack of baseline BP and/or UPCR data, not taking RAS inhibitors before starting Sac/Val, or discontinuation of Sac/Val within 6 months (Figure S1). Clinical characteristics, such as age, sex, etiology of kidney disease, medication use, and laboratory data (e.g., proteinuria and eGFR), were extracted from the electronic medical records. The eGFR was calculated using a population‐specific equation for Japanese patients as follows:

$$\begin{array}{ccc} & & \mathrm{eGFR}\left(\mathrm{mL}/\min/1.73m^{2}\right)=194\;\times \;{\left[\mathrm{age}\left(\mathrm{years}\right)\right]}^{-0.287}\;\\ & & \;\times \;{\left[\mathrm{serum}\mathrm{creatinine}\left(\mathrm{mg}/\mathrm{dL}\right)\right]}^{-1.904}\;\times \;0.739\left(\mathrm{for}\mathrm{females}\right) \end{array}$$

BP, serum potassium concentrations, and the UPCR were assessed immediately before and after 1, 3, and 6 months of Sac/Val treatment. The UPCR was assessed using spot urine samples. The initial dose of Sac/Val and any adjustments to concomitant medications, including antihypertensive agents, were determined at the discretion of the attending physician.

To standardize the doses of different types of RAS inhibitors, the maximum dose of each was set at 100%. An example of this standardization is that because the maximum dose of valsartan in Japan is 160 mg/day, 200 mg of Sac/Val contains 102.8 mg of valsartan, which corresponds to 64.3% of the maximum dose of valsartan as an RAS inhibitor. The maximum doses for each drug in Japan are as follows: valsartan 160 mg/day, irbesartan 200 mg/day, azilsartan 40 mg/day, losartan 100 mg/day, olmesartan 40 mg/day, telmisartan 80 mg/day, and enalapril 10 mg/day.

### 2.2 Statistical Analyses

The results are presented as the mean ± standard deviation, standard error, or the median (lower quartile, upper quartile). Data were analyzed using the paired *t*‐test, independent *t*‐test, Spearman's rank correlation coefficients, the Wilcoxon signed‐rank test, the Mann–Whitney *U*‐test, repeated‐measures analysis of variance, or the Friedman chi‐square test, as appropriate.

Statistical analyses were performed using the SAS Enterprise Guide (version 9.4; SAS Institute, Cary, NC, USA) and EZR (version 1.60; Saitama Medical Center, Jichi Medical University, Saitama, Japan), which is a graphical user interface for R version 4.02 (The R Foundation for Statistical Computing, Vienna, Austria). Statistical significance was set at *p* < 0.05.

## 3 Results

### 3.1 Baseline Clinical Characteristics of the Patients

Blood and urine sampling was conducted at 1, 3, and 6 months after treatment, with a median evaluation period of 35 days (interquartile range [IQR]: 29, 49), 91 days (IQR: 84, 105), and 175 days (IQR: 161, 189), respectively. The mean age of the patients was 65 ± 16 years. The median eGFR and UPCR were 28.4 mL/min/1.73 m^2^ (IQR: 21.1, 44.5) and 1.18 g/g (IQR: 0.44, 3.13), respectively. A total of 55% of the patients had advanced CKD, which was defined as an eGFR < 30 mL/min/1.73 m^2^. According to CKD staging, the numbers of patients in stages G1, G2, G3a, G3b, G4, and G5 were 2, 6, 9, 13, 29, and 7, respectively. The causes of kidney disease included diabetic kidney disease (*n* = 19), hypertensive nephrosclerosis (*n* = 12), chronic glomerulonephritis (*n* = 9), polycystic kidney disease (*n* = 8), and others (*n* = 18).

### 3.2 Changes in Clinical Parameters After Sac/Val Administration

The clinical characteristics of patients before and after Sac/Val treatment are shown in Table 1. Systolic and diastolic BP were significantly reduced after Sac/Val treatment compared with baseline (*p* < 0.0001 and *p* = 0.01, respectively), despite the number of prescribed antihypertensive medications remaining unchanged after switching to Sac/Val at 6 months. Additionally, the eGFR and UPCR were significantly decreased after Sac/Val treatment compared with baseline (*p* = 0.001 and *p* = 0.03, respectively), but this magnitude did not appear to be clinically relevant. Serum potassium concentrations remained stable in these patients.

**TABLE 1:** Characteristics of the patients and changes in clinical parameters after Sac/Val treatment (*n* = 61).

	Baseline	After 1 month of Sac/Val treatment	After 3 months of Sac/Val treatment	After 6 months of Sac/Val treatment	*p*
Systolic BP (mmHg)	144 ± 16	137 ± 19^*^	133 ± 18^***^	135 ± 18^**^	<0.0001
Diastolic BP (mmHg)	78 ± 14	75 ± 15	73 ± 16^**^	74 ± 13	0.01
eGFR (mL/min/1.73 m^2^)	36.2 ± 22.6	36.1 ± 22.5	35.3 ± 22.4	34.8 ± 23.4	0.001
UPCR (g/g)	1.16 (0.50, 3.13)	1.4 (0.55, 2.95)	1.25 (0.41, 2.85)	1.13 (0.36, 2.09)^†^	0.03
Potassium (mmol/L)	4.6 ± 0.5	4.6 ± 0.5	4.7 ± 0.5	4.7 ± 0.5	0.89
Standardized dose of RAS inhibitor (%)	75 ± 29	64 ± 32	74 ± 42	81 ± 39^††^	0.004
Number of antihypertensive drugs	3.3 ± 1.5	3.1 ± 1.3	3.1 ± 1.4	3.1 ± 1.3	0.20
RAS inhibitor, *n* (%)	61 (100)	9 (14.8)	10 (16.4)	8 (13.1)	<0.0001
Calcium channel blocker, *n* (%)	53 (86.9)	50 (82.0)	49 (80.3)	55 (90.2)	0.04
Mineralocorticoid receptor antagonist *n* (%)	16 (26.2)	15 (24.6)	15 (24.6)	13 (21.3)	0.29
*β*‐Blocker, *n* (%)	22 (36.1)	20 (32.8)	20 (32.8)	20 (32.8)	0.39
*α*‐Blocker, *n* (%)	18 (29.5)	16 (26.2)	15 (24.6)	13 (21.3)	0.13
Methyldopa, *n* (%)	3 (4.9)	2 (3.3)	3 (4.9)	3 (4.9)	0.80
Diuretic, *n* (%)	21 (34.4)	15 (24.6)	19 (31.1)	17 (27.9)	0.12
SGLT2 inhibitor, *n* (%)	20 (32.8)	19 (31.1)	24 (39.3)	23 (37.7)	0.06

*Note*: Data are presented as the mean ± standard deviation, median (25th and 75th percentiles), or *n* (%).

Abbreviations: BP, blood pressure; eGFR, estimated glomerular filtration rate; RAS, renin–angiotensin system; Sac/Val, sacubitril/valsartan; SGLT, sodium–glucose cotransporter; UPCR, urinary protein‐to‐creatinine ratio. Data were analyzed by repeated‐measures analysis of variance for normally distributed variables and the Friedman chi‐square test for non‐normally distributed variables.

* *p* < 0.05.

** *p* < 0.01.

*** *p* < 0.001 versus baseline.

^†^ *p* < 0.05.

^††^ *p* < 0.01 versus after 1 month of treatment. *p* values were adjusted by the Bonferroni method.

No substantial changes were observed in the overall use of antihypertensive medications other than RAS inhibitors or in the prescription rates of sodium–glucose cotransporter 2 (SGLT2) inhibitors, both of which can affect proteinuria. The use of calcium channel blockers was significantly increased at 6 months after Sac/Val treatment compared with baseline (*p* = 0.04).

### 3.3 Associations Between the Percent Change in the UPCR, the eGFR, and Systolic BP 1 Month Following Sac/Val Administration

**FIGURE 1:**
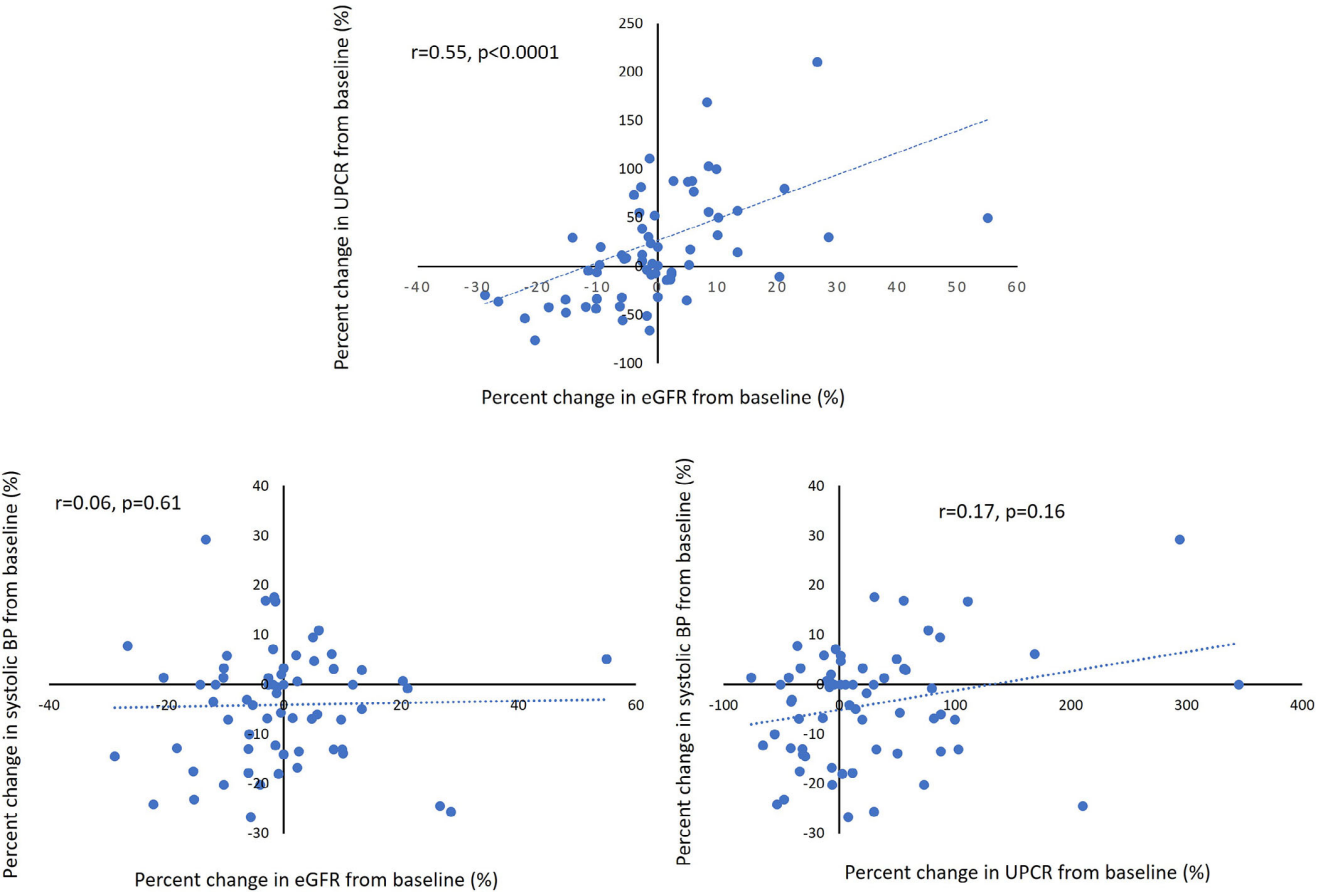
Correlations between the percent change in the UPCR, the eGFR, and systolic BP from baseline. There was a positive correlation between the percent change in the UPCR and the percent change in the eGFR from baseline to 1 month after treatment. BP, blood pressure; eGFR, estimated glomerular filtration rate; UPCR, urinary protein‐to‐creatinine ratio. Spearman's rank correlation coefficients are shown.

Systolic and diastolic BP was significantly decreased at 1 month after Sac/Val treatment compared with baseline (*p* = 0.003 and *p* = 0.02, respectively). Additionally, the number of prescribed antihypertensive medications was significantly decreased at 1 month after Sac/Val treatment compared with baseline (*p* = 0.046, Table S1). The percent change in the UPCR after 1 month was positively correlated with the percent change in the eGFR (*r* = 0.55, *p* < 0.0001, Figure 1). However, there was no association between the percent change in systolic BP and the eGFR (*r* = 0.06, *p* = 0.61) or the UPCR (*r* = 0.17, *p* = 0.16) after 1 month of treatment.

### 3.4 Changes in the UPCR After Sac/Val Treatment

**FIGURE 2:**
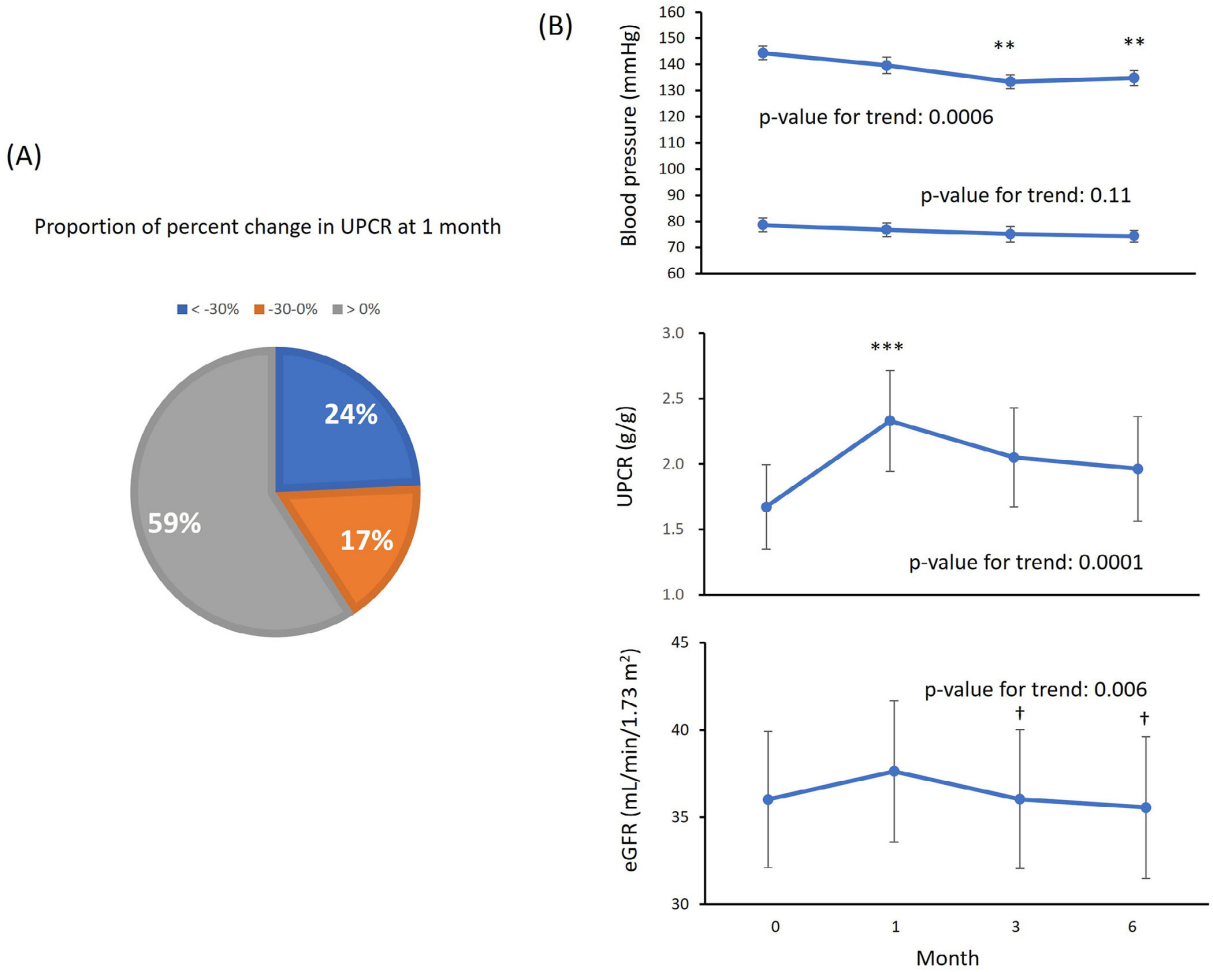
Effect of Sac/Val on proteinuria. (A) Proportion of the percent change in the UPCR from baseline to 1 month of treatment (*n* = 66). (B) The trajectory of BP, the UPCR, and the eGFR in patients with an increased UPCR after treatment with Sac/Val (*n* = 35). Data are shown as the mean ± standard error. **p < 0.01, ***p < 0.001 versus baseline; †*p* < 0.05 versus 1 month after Sac/Val treatment. *p* values were adjusted by the Bonferroni method. BP, blood pressure; eGFR, estimated glomerular filtration rate; UPCR, urinary protein‐to‐creatinine ratio.

At 1 month after Sac/Val treatment, 59% of patients showed an increase in the UPCR, while 24% had a ≥30% decrease in the UPCR (Figure 2A). The patients were divided into two groups according to whether the UPCR increased or decreased, and clinical parameters were evaluated (Table 2). In the group with a decrease in the UPCR, systolic and diastolic BP was significantly decreased (*p* = 0.002 and *p* = 0.01, respectively) and the eGFR was significantly decreased (mean change: −7.1%, *p* = 0.003) at 1 month compared with baseline. By contrast, in the group with an increase in the UPCR, the eGFR was significantly increased at 1 month compared with baseline (mean change: +4.3%, *p* = 0.04). Among the 39 patients who showed an increased UPCR at 1 month, follow‐up data were available for 35 patients at 3 and 6 months (Figure 2B). The increase in the UPCR was observed only in the first month, and it tended to return to baseline levels thereafter (Figure 2B). The standardized RAS inhibitor dose tended to be decreased after 1 month, but there was no significant difference compared with baseline (Table 2). The prescription rates of mineralocorticoid receptor antagonists, diuretics, and SGLT2 inhibitors (agents known to affect proteinuria) did not significantly change from baseline to 1 month in either UPCR group. Additionally, the baseline prescription rates of these medications were similar in the two groups.

**TABLE 2:** Characteristics of the patients and changes in clinical parameters from baseline to 1 month after Sac/Val treatment based on changes in the UPCR.

	Change in the UPCR						
	Decrease < 0% (*n* = 27)			Increase ≥ 0% (*n* = 39)			
	Baseline	After 1 month of Sac/Val treatment	*p*	Baseline	After 1 month of Sac/Val treatment	*p*	*p* ^*^
Systolic BP (mmHg)	143 ± 17	134 ± 20	0.002	145 ± 17	140 ± 18	0.13	0.67
Diastolic BP (mmHg)	76 ± 12	72 ± 12	0.01	79 ± 15	77 ± 16	0.21	0.39
eGFR (mL/min/1.73 m^2^)	36.5 ± 22.0	33.8 ± 20.2	0.003	34.3 ± 22.8	35.7 ± 23.7	0.04	0.93
UPCR (g/g)	1.78 (0.42, 4.97)	0.93 (0.24, 4.34)	< 0.0001	1.14 (0.44, 2.08)	1.50 (0.92, 2.55)	< 0.0001	0.21
Standardized dose of RAS inhibitor (%)	78 ± 31	66 ± 32	0.09	74 ± 28	64 ± 32	0.10	0.62
Number of antihypertensive drugs	3.6 ± 1.3	3.4 ± 1.1	0.21	3.1 ± 1.6	2.9 ± 1.4	0.12	0.26
Mineralocorticoid receptor antagonist, *n* (%)	7 (25.9)	7 (25.9)	1.00	11 (28.2)	10 (25.6)	0.32	0.84
Diuretic, *n* (%)	12 (44.4)	10 (37.0)	0.16	11 (28.2)	7 (17.9)	0.10	0.18
SGLT2 inhibitor, *n* (%)	12 (44.4)	11 (40.7)	0.57	9 (23.1)	9 (23.1)	1.00	0.07

*Note*: Data are presented as the mean ± standard deviation median (25th and 75th percentile), or *n* (%). Abbreviations used in this table are the same as in Table 1.

* Comparison between the two groups at baseline using the *t*‐test for normally distributed variables and the Mann–Whitney *U*‐test for non‐normally distributed variables.

When patients were stratified according to the baseline eGFR (≥30 or <30 mL/min/1.73 m^2^), Sac/Val treatment did not significantly affect proteinuria in either group at 1 month (Table S2).

### 3.5 Changes in the eGFR After Treatment With Sac/Val

**FIGURE 3:**
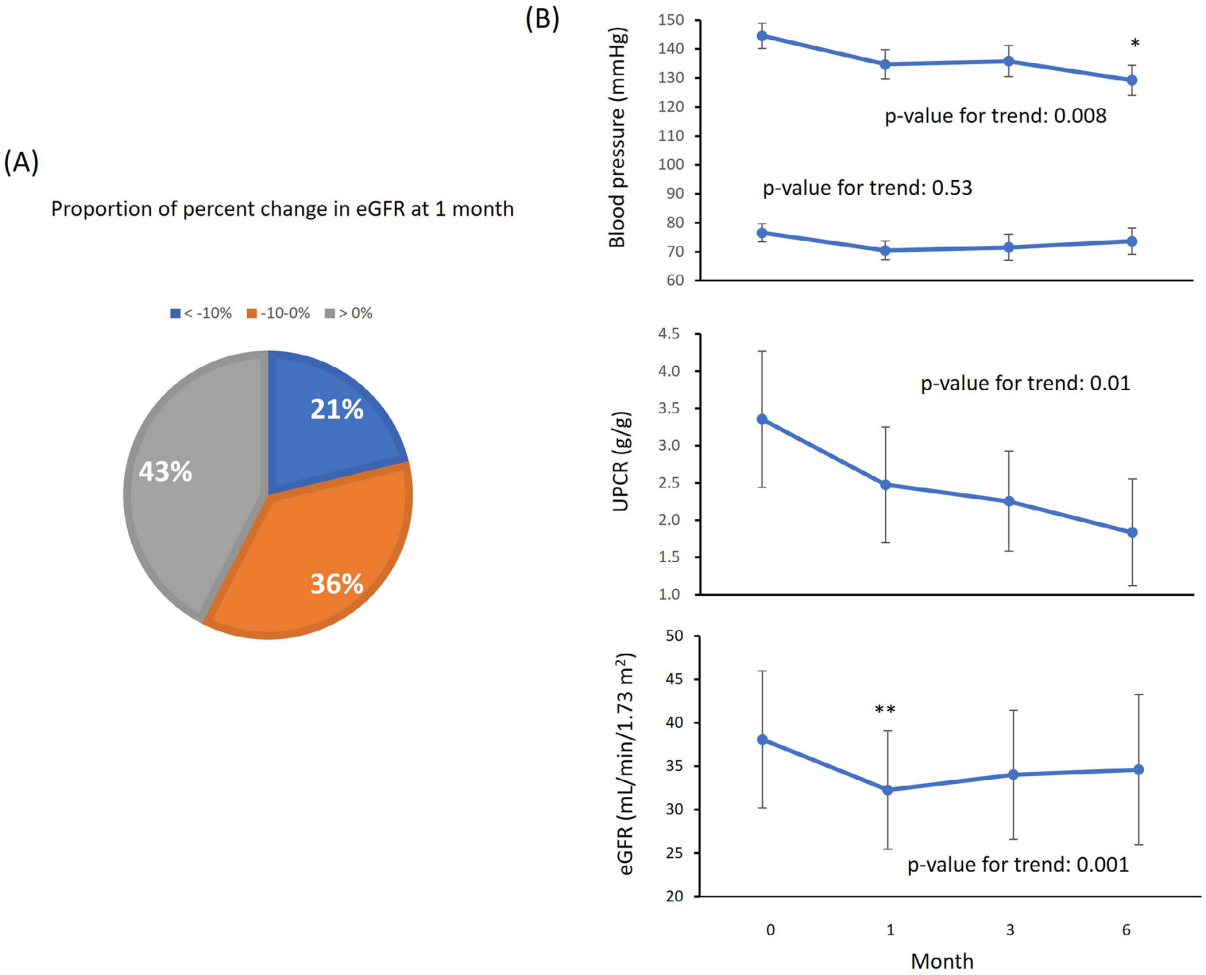
Effect of Sac/Val on the eGFR. (A) Proportion of the percent change in the eGFR from baseline to 1 month of Sac/Val treatment (*n* = 66). (B) The trajectory of BP, the UPCR, and the eGFR in patients with a ≥ 10% decline in the eGFR from baseline to 1 month after initiating Sac/Val (*n* = 12). Data are shown as the mean ± standard error. *p < 0.05, **p < 0.01 versus baseline. *p* values were adjusted by the Bonferroni method. BP, blood pressure; eGFR, estimated glomerular filtration rate; UPCR, urinary protein‐to‐creatinine ratio.

At 1 month, 43% of patients showed an increase in the eGFR, while 21% experienced a ≥10% decline from baseline (Figure 3A). When we compared the groups at baseline, patients with a ≥10% decline in the eGFR were prescribed a greater number of antihypertensive medications than those with a <10% decline in the eGFR (*p* = 0.03). Additionally, baseline use of diuretics was significantly more frequent in patients in the ≥10% eGFR decline group than in those in the <10% eGFR decline group (*p* = 0.009). The group of patients with a ≥10% decline in the eGFR tended to have a higher UPCR than those with a <10% decline, but this difference was not significant (*p* = 0.16) (Table 3).

**TABLE 3:** Characteristics of the patients and changes in clinical parameters from baseline to after 1 month of Sac/Val treatment based on changes in the eGFR.

	Change in eGFR						
	≥10% decline (*n* = 14)			No decline or <10% decline (*n* = 52)		
	Baseline	After 1 month of Sac/Val treatment	*p*	Baseline	After 1 month of Sac/Val treatment	*p*	*p* ^*^
Systolic BP (mmHg)	143 ± 16	135 ± 18	0.13	144 ± 17	139 ± 19	0.01	0.86
Diastolic BP (mmHg)	75 ± 11	71 ± 10	0.09	79 ± 15	76 ± 15	0.07	0.47
eGFR (mL/min/1.73 m^2^)	36.5 ± 25.9	30.7 ± 22.3	0.0001	34.9 ± 21.5	36.1 ± 22.3	0.04	0.62
UPCR (g/g)	2.28 (0.59, 4.49)	1.33 (0.64, 2.95)	0.02	1.06 (0.40, 2.24)	1.45 (0.48, 2.74)	0.002	0.16
Standardized dose of RAS inhibitor (%)	84 ± 33	80 ± 39	0.66	73 ± 28	61 ± 28	0.02	0.23
Number of antihypertensive drugs	4.1 ± 1.5	3.9 ± 1.3	0.34	3.1 ± 1.4	2.9 ± 1.3	0.09	0.03
Mineralocorticoid receptor antagonist, *n* (%)	5 (35.7)	5 (35.7)	1.00	13 (25.0)	12 (23.1)	0.32	0.43
Diuretic, *n* (%)	9 (64.3)	8 (57.1)	0.36	14 (26.9)	9 (17.3)	0.06	0.009
SGLT2 inhibitor, *n* (%)	4 (28.6)	4 (28.6)	1.00	17 (32.7)	16 (30.8)	0.57	0.77

*Note*: Data are presented as the mean ± standard deviation, median (25th and 75th percentile), or *n* (%).Abbreviations used in this table are the same as in Table 1.

* Comparison between the two groups at baseline using the *t*‐test for normally distributed variables, and the Mann–Whitney *U*‐test for non‐normally distributed variables.

We also compared the change in variables between baseline and 1 month after Sac/Val treatment within the ≥10% eGFR decline and <10% eGFR decline groups. In the ≥10% eGFR decline group, the UPCR significantly decreased at 1 month compared with baseline (*p* = 0.02), whereas it was significantly increased in the <10% eGFR decline group (*p* = 0.002). The standardized dose of RAS inhibitors remained unchanged in the ≥10% eGFR decline group but was decreased in the <10% eGFR decline group at 1 month (*p* = 0.02). Among the 14 patients with a ≥10% decline in the eGFR, follow‐up data for 12 patients at 3 and 6 months showed a trend toward reductions in systolic BP and the UPCR over time (Figure 3B). Notably, the eGFR returned closer to baseline levels after the initial decline.

When patients were stratified according to the baseline eGFR (≥30 or <30 mL/min/1.73 m^2^), Sac/Val treatment did not significantly affect the eGFR in either group at 1 month (Table S2).

## 4 Discussion

In this study, treatment with Sac/Val led to a significant and sustained reduction in systolic BP from 1 to 6 months. While reductions in proteinuria and eGFR were also observed, these changes were modest and not clinically significant. A positive correlation between changes in the eGFR and UPCR at 1 month was identified. Notably, patients who experienced an early decline in the eGFR 1 month after treatment showed a progressive reduction in proteinuria, whereas those with an increase in the UPCR at 1 month showed a return toward baseline proteinuria levels, although they were still elevated.

Sac/Val induces dilation of the afferent arterioles through the action of NPs. NPs promote the relaxation of mesangial cells, thereby expanding the filtration surface area and increasing the glomerular filtration coefficient. Furthermore, through valsartan's inhibition of angiotensin II action, Sac/Val causes dilation of the efferent arterioles [12]. These combined effects are thought to suppress the increase in intraglomerular pressure. Consequently, Sac/Val is expected to lower intraglomerular pressure while preserving the GFR. Even in patients with CKD and a low or normal absolute GFR, relative glomerular hyperfiltration can persist. RAS and SGLT2 inhibitors reduce intraglomerular pressure by efferent arteriolar dilation and restoration of tubuloglomerular feedback mechanisms, respectively, thereby contributing to renoprotection. However, not all patients experience an initial decline in the eGFR. Some patients show an increase in the eGFR, likely reflecting individual differences in the underlying pathophysiology [13, 14, 15]. In this study, 43% of patients showed an increase in the eGFR at 1 month, despite no change in the background doses of RAS inhibitors. This finding occurred despite a significant reduction in systolic BP at 1‐month post‐treatment of Sac/Val compared with baseline. This finding suggested that increased NPs, a known effect of Sac/Val, may have induced afferent arteriolar dilation, thereby contributing to an elevated eGFR and increased proteinuria.

In contrast, 21% of patients experienced a ≥10% decline in the eGFR. These patients tended to have higher baseline proteinuria, which suggested elevated intraglomerular pressure, and they required more antihypertensive agents. The higher prescription rate of diuretics in these patients may also have increased susceptibility to the decline in the eGFR. Furthermore, a reduction in proteinuria at 1 month after Sac/Val treatment was more closely associated with the decline in the eGFR than with the reduction in BP, suggesting that Sac/Val provides nephroprotection in selected patients via modulation of intraglomerular hemodynamics. All currently approved and investigational renoprotective agents induce an initial decline in the eGFR, supporting an association between glomerular hyperfiltration and CKD progression [16]. Longitudinal data from a large Taiwanese cohort treated with SGLT2 inhibitors showed that an early simultaneous reduction in the eGFR and UACR was associated with a lower risk of renal outcomes, whereas a decline in the eGFR without an improvement in the UACR was associated with poorer outcomes [17]. These findings support the concept that dual early reductions in eGFR and proteinuria may represent favorable hemodynamic responses.

The effect of Sac/Val on proteinuria remains inconsistent across studies [9, 10, 11]. Although Sac/Val is associated with increased albuminuria compared with RAS inhibitors, the PARADIGM‐HF trial suggested that Sac/Val provides renoprotective effects in patients with HF [4]. Moreover, the inhibition of neutral endopeptidase has been reported to increase the eGFR, potentially leading to increased proteinuria following Sac/Val administration [4, 18]. In contrast, the UK HARP‐III trial [10] showed that while Sac/Val significantly lowered BP in patients with CKD (*p* < 0.001), it was associated with a nonsignificant 9% reduction in the UACR compared with an RAS inhibitor. Additionally, this reduction appeared to be associated with a lowering of BP. Previous studies have reported that Sac/Val therapy reduces proteinuria or albuminuria in patients with advanced CKD, after 6 months in association with a reduction in BP or after 8 weeks in patients with higher baseline albuminuria [9, 19]. In the present study, proteinuria was increased in 59% of patients at 1 month after Sac/Val treatment was started, but returned to near baseline levels by 3–6 months, along with reductions in BP and the eGFR. These findings suggest that a reduction in BP may partly contribute to the decrease in proteinuria in patients with CKD.

Sac/Val is also associated with fewer cases of hyperkalemia and elevated creatinine concentrations, leading to treatment discontinuation, compared with RAS inhibitors [20, 21]. Several studies have reported that Sac/Val can be safely administered even in patients with CKD [11, 19]. In this study, more than half of the patients who switched to or added Sac/Val to ongoing RAS inhibitor therapy had an eGFR < 30 mL/min/1.73 m^2^. Despite this finding, Sac/Val significantly reduced BP without causing a clinically significant decline in the eGFR or incidence of hyperkalemia.

Further studies are warranted to evaluate the long‐term renal and cardiovascular effects of Sac/Val to determine the mechanisms underlying its effect on the eGFR and proteinuria, and to identify patient populations most likely to benefit from its use.

## 5 Limitations

This study has several limitations. Treatment decisions were at the discretion of attending physicians, which caused difficulty in isolating the BP‐lowering effect of Sac/Val. The discontinuation of diuretics or mineralocorticoid receptor antagonists may have affected the proteinuria results. This study was retrospective, lacked a control group, and included a small, single‐center sample, which limited the generalizability of the findings. Additionally, differences in RAS inhibitor dosing across countries may have limited the accuracy of RAS inhibitor dose conversion. Larger, long‐term, controlled studies are required to better characterize the clinical profiles of patients who benefit most from Sac/Val therapy.

## 6 Conclusion

Sac/Val effectively reduces BP in patients with hypertension and advanced CKD without accelerating a decline in the eGFR or causing hyperkalemia. However, the effects of Sac/Val on proteinuria vary and may be more pronounced in patients with higher baseline proteinuria.

## Author Contributions

M.M. and T.G. contributed to the study design, data analysis, and manuscript writing. T.K., M.K., S.U., and Y.S. provided critical intellectual input during manuscript development. TG oversaw all aspects of the research and analysis. M.M. prepared the manuscript for submission. All authors read and approved the final manuscript.

## Ethics Statement

This was an opt‐out study. This study was approved by the Ethics Committee of Juntendo University Hospital, with a waiver of the requirement to obtain informed consent (approval number: E24‐0175‐H01), and was conducted in accordance with the 1964 Helsinki declaration and its later amendments or comparable ethical standards.

## Conflicts of Interest

Y.S. received honoraria from Novartis and Otsuka Pharmaceuticals.

## Supporting information




**Supporting File**: Supplementary Figure 1 Flowchart representing the inclusion criteria for patients in this study. The effect of sacubitril/valsartan was ultimately evaluated in 66 patients with hypertension and proteinuria.

## Data Availability

The datasets generated during and/or analyzed during the current study are available from the corresponding author upon reasonable request.
