# Supplementary material for: Effects of Sacubitril/Valsartan on Blood Pressure and Proteinuria in Hypertensive Patients With Chronic Kidney Disease
Source: J Clin Hypertens (Greenwich). 2025 Jul 20;27(7):e70089. doi: 10.1111/jch.70089 (PMC12277533; doi:10.1111/jch.70089)
Supplement: Supplementary file 1 — Supporting File: Supplementary Figure 1 Flowchart representing the inclusion criteria for patients in this study. The effect of sacubitril/valsartan was ultimately evaluated in 66 patients with hypertension and proteinuria. [file JCH-27-e70089-s001.pdf]

Supplementary Figure 1. Flowchart representing the inclusion criteria for patients in this study.

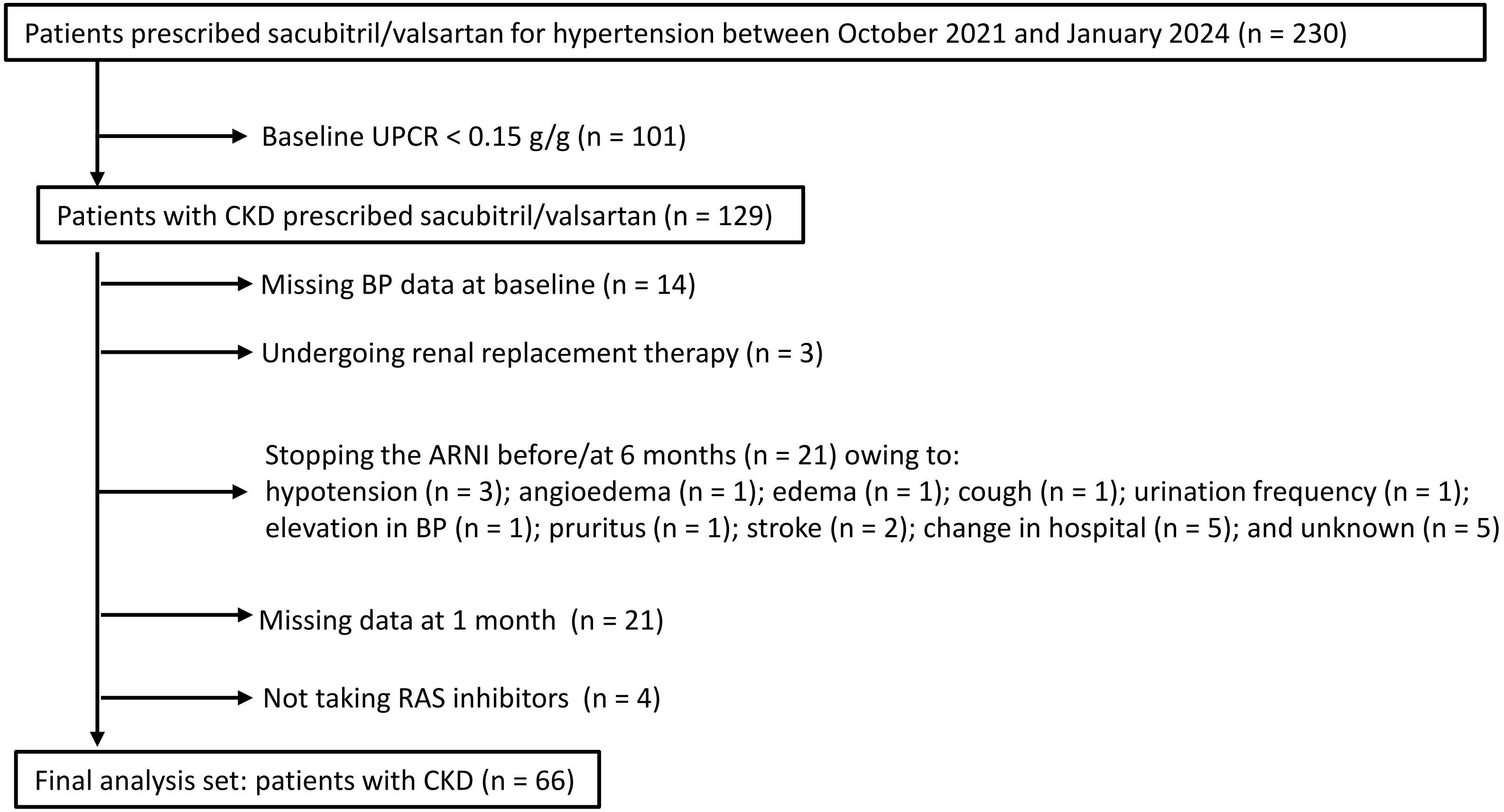

Supplementary Table 1. Classes of antihypertensive medications in patients who were treated by RAS inhibitors at baseline and 1 month Sac/Val treatment

Medication (n=66)	Baseline	After 1 month of Sac/Val treatment
Number of antihypertensive drugs	3.3 ± 1.5	3.1 ± 1.3
RAS inhibitor	66 (100)	9 (13.6)
Calcium channel blocker	56 (84.8)	54 (81.8)
Mineralocorticoid receptor antagonist	18 (27.3)	17 (25.8)
β-blocker	23 (34.8)	21 (31.8)
α-blocker	20 (30.0)	17 (25.8)
Methyldopa	4 (6.1)	3 (4.5)
Diuretic	23 (34.8)	17 (25.8)

Data are presented as the mean ± standard deviation, or n (%).

Abbreviations: RAS, renin–angiotensin system; Sac/Val, sacubitril/valsartan.

Supplementary Table 2. Characteristics of patients and changes in clinical parameters after Sac/Val treatment (n = 66)

	Baseline	After 1 month of Sac/Val treatment	P
eGFR > 30mL/min/1.73 m^2 (n = 29)			
Systolic BP (mmHg)	142 ± 17	135 ± 20	0.02
Diastolic BP (mmHg)	80 ± 13	77 ± 14	0.07
eGFR (mL/min/1.73 m^2)	52.9 ± 22.9	52.2 ± 22.9	0.78
UPCR (g/g)	0.90 (0.32, 1.78)	0.99 (0.26, 2.06)	0.35
Potassium (mmol/L)	4.4 ± 0.4	4.4 ± 0.3	0.90
eGFR < 30mL/min/1.73 m^2 (n = 37)			
Systolic BP (mmHg)	146 ± 16	140 ± 18	0.047
Diastolic BP (mmHg)	76 ± 15	73 ± 15	0.09
eGFR (mL/min/1.73 m^2)	21.3 ± 6.4	21.5 ± 7.6	0.54
UPCR (g/g)	1.52 (0.66, 4.28)	1.53 (0.92, 4.55)	0.48
Potassium (mmol/L)	4.8 ± 0.6	4.8 ± 0.5	0.94

Data are presented as mean ± standard deviation, or median (25th and 75th percentile).

Abbreviations: BP, blood pressure; eGFR, estimated glomerular filtration rate; Sac/Val, sacubitril/valsartan; UPCR, urinary protein-to-creatinine ratio.
